# Conversion surgery for an initially unresectable, locally advanced pancreatic cancer after induction chemotherapy and carbon-ion radiotherapy: a case report

**DOI:** 10.1186/s40792-018-0522-4

**Published:** 2018-09-10

**Authors:** Takeshi Fujishiro, Taro Mashiko, Yosihito Masuoka, Misuzu Yamada, Daisuke Furukawa, Naoki Yazawa, Yohei Kawashima, Masami Ogawa, Kenichi Hirabayashi, Toshio Nakagohri

**Affiliations:** 1 Department of Surgery, Tokai University School of Medicine, 143 Shimokasuya, Isehara-shi, Kanagawa 259-1193 Japan; 2 Department of Gastroenterology, Tokai University School of Medicine, 143 Shimokasuya, Isehara-shi, Kanagawa 259-1193 Japan; 3 Department of Pathology, Tokai University School of Medicine, 143 Shimokasuya, Isehara-shi, Kanagawa 259-1193 Japan

**Keywords:** Locally advanced pancreatic cancer, Carbon-ion radiotherapy, Gemcitabine + nab-paclitaxel, Conversion surgery

## Abstract

**Background:**

Pancreatic cancer has a very high mortality rate worldwide, and about 30–40% of all patients have extensive vascular involvement at initial diagnosis that precludes surgical intervention. Here, we describe our experience in a patient with locally advanced pancreatic cancer (LAPC) who underwent R0 conversion surgery after undergoing a combination of chemotherapy and carbon-ion radiotherapy (CIRT), which led to long-term relapse-free survival (23 months).

**Case presentation:**

A 41-year-old woman presented a month ago with epigastralgia referred to our facility and was subsequently diagnosed with pancreatic cancer cStage III (Ph, TS2 (35 mm), cT4, cCH1, cDU1, cS1, cRP1, cPL1, cVsm0, cAsm1, cN0, cM0) that was also categorized as an unresectable LAPC. She immediately underwent 3 cycles of induction chemotherapy (gemcitabine + nanoparticle albumin-bound (nab-) paclitaxel) followed by CIRT with concurrent gemcitabine. Although significant shrinkage of the primary tumor occurred, frequent cholangitis due to duodenal stenosis of unknown etiology prevented continued chemotherapy, and 9 months after the first visit, she underwent a radical, subtotal, stomach-preserving, pancreaticoduodenectomy (SSPPD). Histopathologic examination of the resected tissue revealed a R0 resection with a histological response of Evans grade IIB. She was administered an almost full dose of S-1 as adjuvant chemotherapy for 6 months and has shown no signs of recurrence in 23 months.

**Conclusions:**

We report a first case of successful conversion surgery for an initially unresectable LAPC after rapid induction GEM + nab-PTX chemotherapy followed by CIRT. Rapid induction GEM + nab-PTX chemotherapy followed by CIRT for LAPC might be a safe and effective treatment option.

## Background

In Japan, the number of deaths due to pancreatic cancer have risen sharply (> 30,000 cases/year), and 30–40% of these patients have extensive vascular involvement at initial diagnosis that precludes surgical intervention [1–3]. Locally advanced pancreatic cancer (LAPC) is defined as unresectable pancreatic cancer due to vascular involvement without radiographically distant metastases. Recently, some reports have revealed that induction therapy followed by conversion surgery for LAPC may be more efficacious [2, 4, 5]; however, there is insufficient evidence to support this regimen from randomized phase III trials. Carbon-ion beams are also considered ideal and powerful topical therapy for LAPC because of its biological features. Here, we describe our experience in a patient with LAPC who is currently in long-term relapse-free survival after undergoing conversion surgery after induction chemotherapy followed by CIRT.

## Case presentation

**Fig. 1:**
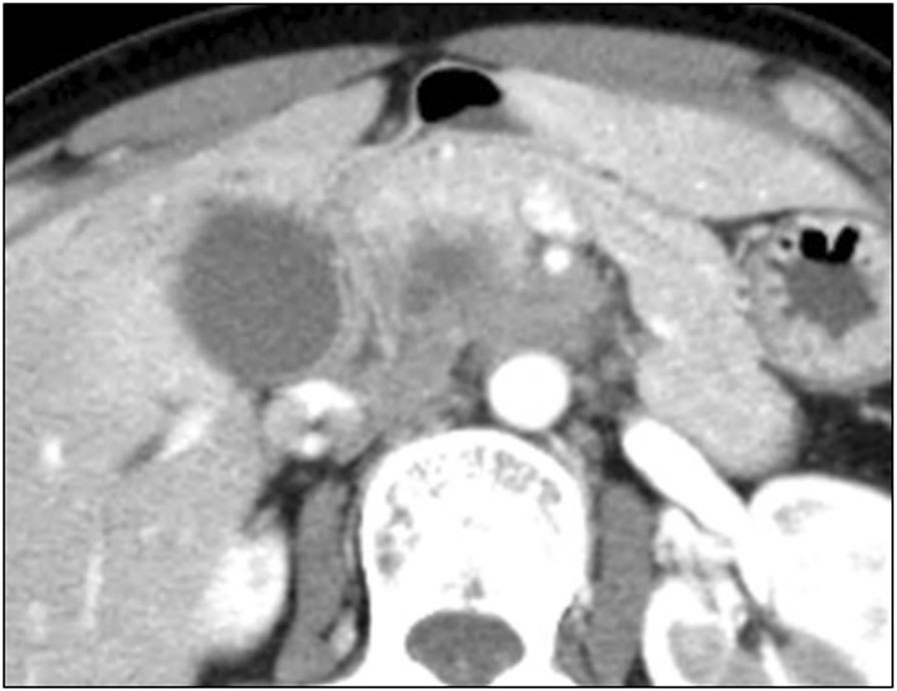
Arterial phase of the MDCT at first visit shows a massive hypovascular tumor in the uncinate process that was in direct contact with the SMA (> 180°), but without stenosis or obstruction. It was clinically diagnosed as an unresectable LAPC (defined by the criteria of JPS, 7th ed.)

A 41-year-old woman presented a month ago with epigastralgia referred to our facility. Physiological and laboratory assessments were unremarkable except for elevated serum CA19-9 and elastase-1 levels (CA19-9, 207 IU/L; elastase-1, 150 IU/L). Enhanced multi-detector CT revealed a hypovascular tumor in the uncinate process of the pancreas that was in wide contact with the superior mesenteric artery (SMA, > 180°; Fig. 1). Endoscopic retrograde cholangiopancreatography (ERCP) demonstrated stenosis of the main pancreatic duct and mild distal dilatation. Pancreatic juice and biliary juice cytology were categorized as class V (adenocarcinoma), and a biliary plastic stent was placed during the initial ERCP procedure. She was subsequently diagnosed with cStage III pancreatic cancer (Ph, TS2 (35 mm), cT4, cCH1, cDU1, cS1, cRP1, cPL1, cVsm0, cAsm1, cN0, cM0) that was also categorized as an unresectable LAPC (UR-LA) according to the Japan Pancreatic Society (JPS) classification, 7th edition [6, 7]. Therefore, she immediately underwent 3 cycles of chemotherapy (gemcitabine (GEM) 1000 mg/m^2^ + nanoparticle albumin-bound paclitaxel (nab-PTX) 260 mg/m^2^) on days 1, 8, and 15 of a 28-day cycle. In addition, she underwent 55.2 GyE (RBE) of CIRT with concurrent GEM chemotherapy (GEM 1000 mg/m^2^, days 1, 8, and 15) after GEM + nab-PTX chemotherapy.

**Fig. 2:**
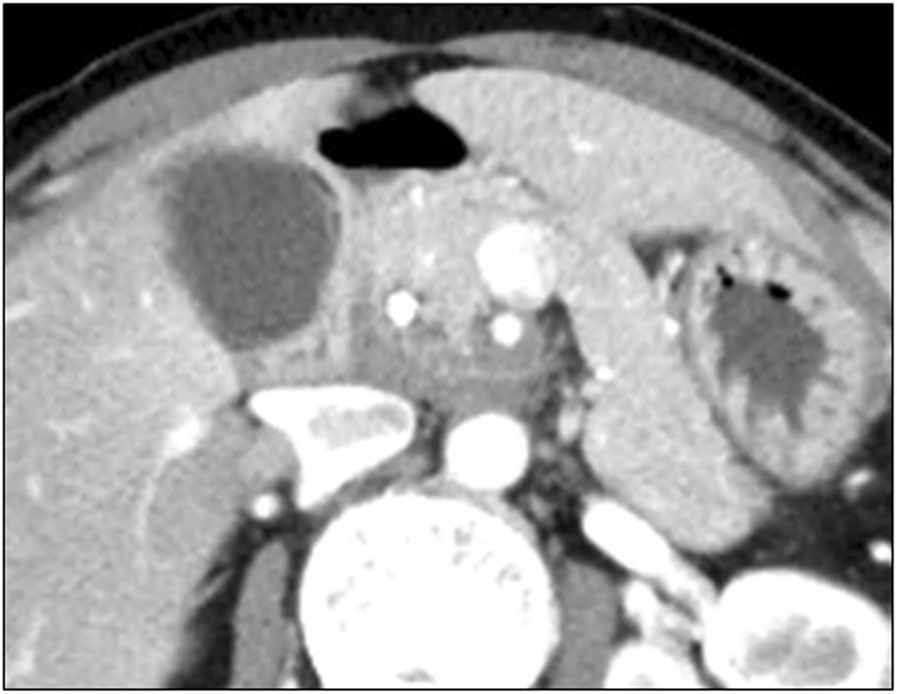
MDCT showed remarkable tumor shrinkage after three courses of induction GEM + nab-PTX chemotherapy. However, the low-density area around the SMA still remained

**Fig. 3:**
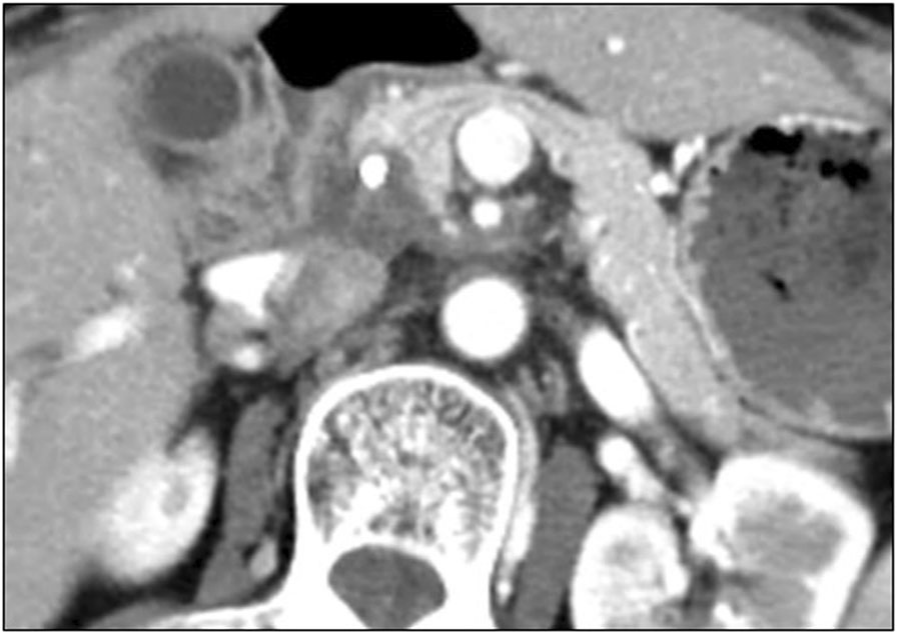
The primary tumor shrank further after CIRT, and it was difficult to evaluate if viable tumor cells were present around the SMA from the MDCT images

**Fig. 4:**
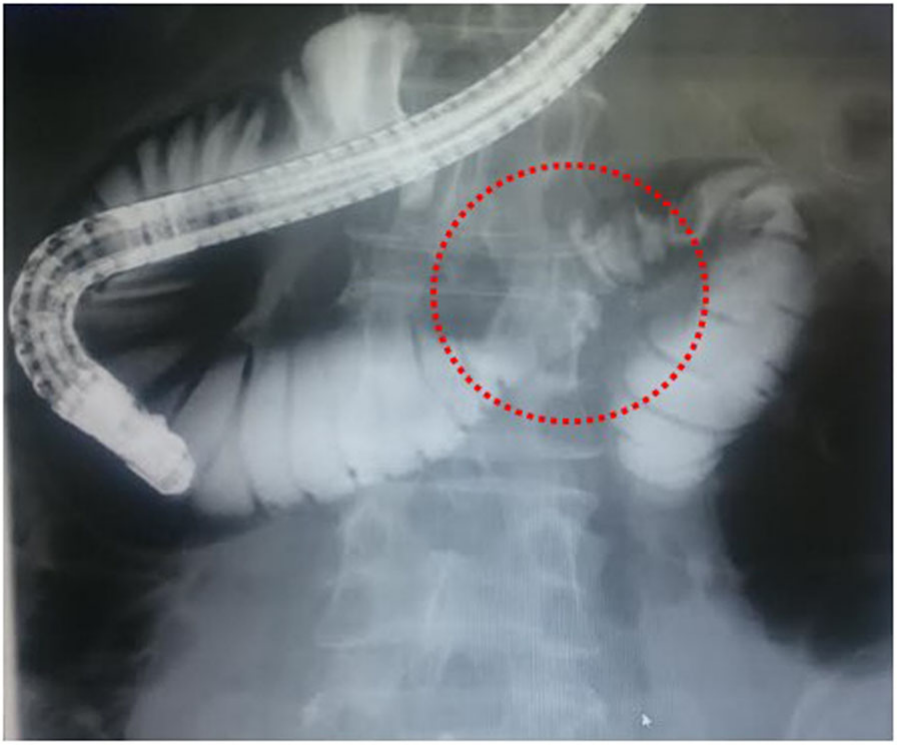
Duodenography, added to ERCP, showed a slight narrowing (red circle) of the third portion and dilatation of the oral side; however, air sent from the endoscope could pass through the narrowed area. Clinically, the patient did not complain of gastrointestinal obstruction before surgery

CA19-9 antigen levels fell within normal range (CA19-9, 207 → 25 IU/L) after three courses of induction GEM + nab-PTX chemotherapy and remained within the normal range (CA19-9, 22.9 IU/L) until conversion surgery. Although the primary tumor showed remarkable shrinkage after induction chemotherapy (Fig. 2) and subsequent CIRT (Fig. 3), clinically and radiologically apparent frequent cholangitis due to unidentified duodenal narrowing (Fig. 4), along with peripheral sensory neuropathy due to the nab-PTX regimen, prevented continued chemotherapy. FDG-PET scan did not reveal any hot spots either in the primary tumor or in the entire body, and 9 months after her first visit, we decided to perform conversion surgery. As dissection around the SMA was necessary for achieving a R0 resection, we initially identified and taped the SMA and histology on rapid frozen sections of a part of the nerve plexus around the SMA confirmed absence of tumor cells. Therefore, we performed a radical subtotal stomach preserving pancreaticoduodenectomy (SSPPD). Group 1 and 2 lymph node dissections (D2) were also performed while predominantly preserving the nerve plexus around the SMA, except for the resected tissue used for rapid frozen section analysis (JPS 7th ed.). The procedure lasted 4 h and 55 min with a blood loss of 587 g. Preoperative therapy did not affect surgical factors such as difficulty of the surgical procedure, blood loss, or operation time, and the patient was discharged on the 22nd postoperative day.

**Fig. 5:**
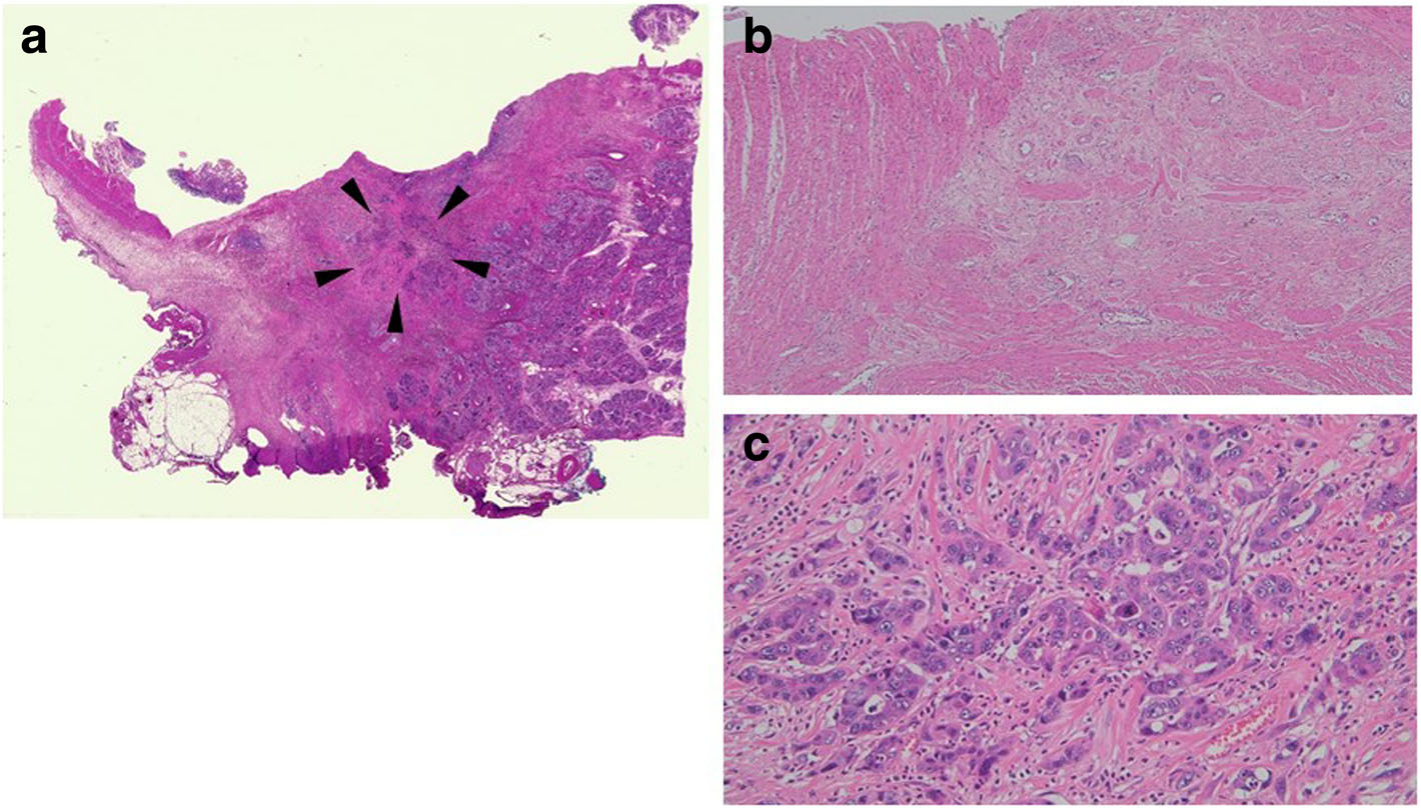
Microscopic findings of the resected specimen. **a** Most of the primary tumor had been replaced by fibrosis, and only a few cancer cells (arrow head) were seen in the central area of the tumor (HE, × 4). **b** The wall structure of the duodenum had also been destroyed and replaced with fibrosis, but there were no cancer cells (HE, × 40). **c** Degenerated cancer cells were visible in the central area of the tumor (EVANS grade IIB, HE,× 200)

Grade B postoperative pancreatic fistula (POPF) and deep incision surgical site infection (SSI) were identified as postoperative complications. Histopathological examination revealed an invasive ductal carcinoma, a tubular adenocarcinoma (tub1>tub2), Ph, TS1(20 × 8 × 7 mm), infiltrative type, ypT3, ypCH1, ypDU0, ypS0, ypRP0, ypPVX, ypAX, ypPL0, ypOO0, ypPCM0, ypBCM0, ypDPM0, intermediate type, INFβ, ly0, v1, ne1 mpd0, ypN0(0/7), ypM0, ypT3N0M0 ypStageIIA. Histologically, it was categorized as Evans grade IIB (Fig. 5) and the patient underwent an almost full dose of S-1 adjuvant chemotherapy for 6 months, and no signs of recurrence have been seen for 23 months.

### Discussion

Pancreatic cancer is associated with an extremely poor prognosis, and prolonged survival is only achieved by R0 resection. However, LAPC accounts for 30–40% of all pancreatic cancers [1–3], and the two internationally used criteria for LAPC are from the National Comprehensive Cancer Network (NCCN) and from the joint consensus conference of the Americas Hepato-Pancreato-Biliary Association (AHPBA), the Society for Surgical Oncology (SSO), and the Society for Surgery of the Alimentary Tract (SSAT) (AHBPA/SSO/SSAT) [8, 9]. JPS has also established its own criteria, and these are followed in Japan [6, 7]. Importantly, it is known that a R0 resection for LAPC cannot be achieved with surgery alone and that LAPC requires powerful local or regional control, including with chemo- and/or radiotherapy. On the other hand, previous reports have shown that one third of all patients with LAPC treated with chemoradiotherapy eventually develop metastatic progression [10–12]. Thus, even in LAPC, it is necessary to deliver more effective systemic chemotherapy early in the course of the cancer, apart from providing locoregional treatment to improve outcomes. The advantages of combining rapid induction chemotherapy and chemoradiotherapy, including carbon-ion beams, are as follows. First, generally, chemotherapy can be introduced rapidly and without major adverse reactions compared to chemoradiotherapy, and second, it can save patients with occult metastasis from undergoing subsequent chemoradiotherapy. Several non-randomized controlled trials have demonstrated the feasibility and efficacy of combining rapid induction chemotherapy and chemoradiation [13, 14]. This therapeutic strategy of “chemotherapy-first” may be considered similar to adjuvant therapy for pancreatic cancer. Recently, more powerful regimens have been made available for pancreatic cancer, and it would be ideal to investigate their efficacy using randomized controlled trial (RCT).

Carbon-ion beams can provide ideal dose distributions [15] and greater biological efficacy [16], and its efficacy does not depend on cell-cycle stage [17]. These biological features are considered as anatomically suitable for locoregional control of LAPC. A few non-RCTs on CIRT for pancreatic cancer are available from Japan [15, 18, 19], and these reports show feasibility and efficacy of CIRT for pancreatic cancer. A recent multi-institutional study on CIRT for LAPC from Japan (J-CROS1403) was conducted in 72 patients, and the results indicate favorable clinical outcomes with median OS of 21.5 months and no severe toxicity-related gastrointestinal disorders, including stenosis or obstruction, except in 1 case (1%) of grade 3 duodenal ulcer due to late toxicity (19). Thus, we believe that it is reasonable to expect that a reduction in the risk of local relapse by CIRT will lead to an increase in the cure rate for LAPC.

In our case, frequent retrograde cholangitis, probably caused by duodenal stenosis, was clinically apparent. Possible explanations include (1) the presence of a huge pancreatic head tumor, due to which the boundary between the tumor and the duodenal wall often remains unclear, or (2) that the tumor has invaded the duodenum. In these scenarios, it is possible that the planned target volume of carbon-ion beams included a part of the duodenum, which then influenced her clinical course. However, the patient did not complain of nausea or bilious vomiting related to duodenal stenosis, and furthermore, pathological examination of resected specimen revealed no obvious evidences of stenosis or direct invasion of the duodenum by the tumor except for mild fibrosis in the muscular layer of the same portion. Thus, the following factors may have caused duodenal compression from outside the duodenal wall. First, while the therapeutic response of grade IIB (EVANS) is an excellent result, the resected specimens showed that most of the tumor had been replaced by fibrosis. Therefore, we cannot discount the possibility that the fibrous tissue inhibited wall ductility of the duodenum. Second, as the patient was thin, it is possible that she had developed the SMA syndrome. Thus, it is our opinion that the retrograde cholangitis was not a simple adverse event of CIRT but the result of a favorable therapeutic response of the tumor.

The duration of induction therapy in this case was 9 months. According to previous reports, the median duration of induction chemo (radio) therapy in patients who undergo conversion surgery is 7–9 months [5, 20, 21]. In our patient, histopathological findings revealed high efficacy of chemoradiotherapy, and the patient is currently in long-term, relapse-free survival. These facts suggest that a combination of rapid induction GEM + nab-PTX chemotherapy followed by CIRT can be safely used and that it might be an effective treatment option for LAPC.

## Conclusions

We report a first case of conversion surgery after a combination of rapid induction GEM + nab-PTX therapy followed by CIRT for an initially unresectable LAPC. The patient is currently undergoing relapse-free survival. The favorable clinical course and outcome suggest that multidisciplinary treatment consisting of a combination of a powerful systemic chemotherapy and a local therapy may be beneficial in LAPC.

## Abbreviations

AHPBA: Americas Hepato-Pancreato-Biliary Association
CIRT: Carbon-ion radiotherapy
CT: Computed tomography
ERCP: Endoscopic retrograde cholangiopancreatography
FDG-PET: 18F-Fluorodeoxyglucose-positron emission tomography
GEM: Gemcitabine
J-CROS: Japan Carbon-ion Radiation Oncology Study Group
JPS: Japan Pancreas Society
LAPC: Locally advanced pancreatic cancer
nab-PTX: Nanoparticle albumin-bound paclitaxel
NCCN: National Comprehensive Cancer Network
OS: Overall survival
POPF: Postoperative pancreatic fistula
RCT: Randomized control trial
SMA: Superior mesenteric artery
SSAT: Society for Surgery of the Alimentary Tract
SSI: Surgical site infection
SSO: Society for Surgical Oncology
SSPPD: Subtotal stomach preserving pancreaticoduodenectomy
